# Pharmacological and Advanced Cell Respiration Effects, Enhanced by Toxic Human-Bile Nano-Pharmaceuticals of Probucol Cell-Targeting Formulations

**DOI:** 10.3390/pharmaceutics12080708

**Published:** 2020-07-29

**Authors:** Susbin Raj Wagle, Bozica Kovacevic, Daniel Walker, Corina Mihaela Ionescu, Melissa Jones, Goran Stojanovic, Sanja Kojic, Armin Mooranian, Hani Al-Salami

**Affiliations:** 1 Biotechnology and Drug Development Research Laboratory, School of Pharmacy and Biomedical Sciences, Curtin Health Innovation Research Institute, Curtin University, Perth WA 6102, Australia; susbinraj.wagle@postgrad.curtin.edu.au (S.R.W.); bozica.kovacevic@postgrad.curtin.edu.au (B.K.); danieljcswalker@gmail.com (D.W.); c.ionescu@postgrad.curtin.edu.au (C.M.I.); melissa.a.jones6@gmail.com (M.J.); a.mooranian@curtin.edu.au (A.M.); 2 Faculty of Technical Sciences, University of Novi Sad, Trg Dositeja Obradovica 6, 21000 Novi Sad, Serbia; sgoran@uns.ac.rs (G.S.); sanjakojic@uns.ac.rs (S.K.)

**Keywords:** bile acids, diabetes mellitus, formulation sciences, inflammation, probucol, unconjugated lithocholic acid

## Abstract

Bile acids have recently been studied for potential applications as formulation excipients and enhancers for drug release; however, some bile acids are not suitable for this application. Unconjugated lithocholic acid (ULCA) has recently shown drug formulation-stabilizing and anti-inflammatory effects. Lipophilic drugs have poor gut absorption after an oral dose, which necessitates the administration of high doses and causes subsequent side effects. Probucol (PB) is a highly lipophilic drug with poor oral absorption that resulted in restrictions on its clinical prescribing. Hence, this study aimed to design new delivery systems for PB using ULCA-based matrices and to test drug formulation, release, temperature, and biological effects. ULCA-based matrices were formulated for PB oral delivery by applying the jet-flow microencapsulation technique using sodium alginate as a polymer. ULCA addition to new PB matrices improved the microcapsule’s stability, drug release *in vitro* (formulation study), and showed a promising effect in *ex vivo* study (*p* < 0.05), suggesting that ULCA can optimize the oral delivery of PB and support its potential application in diabetes treatment.

## 1. Introduction

Diabetes mellitus (DM) is a metabolic disorder characterized by high blood sugar levels resulting in numerous complications and multiple organ damage. Presently, DM is the most common and significant public health problem worldwide, and its prevalence is increasing [1,2]. It is projected that in the year 2030–2040, around 700 million individuals will suffer from DM, and by then, DM will be the seventh leading causes of death, unless new potential and potent drugs are introduced to the market [3,4].

Type one diabetes mellitus (T1DM) is an autoimmune disease resulting in the loss of endogenous insulin production and secretion, while Type 2 DM (T2DM) stems from peripheral insulin resistance and is brought about by genetics and environmental factors such as lifestyle changes, urbanization, aging, obesity, and physical inactivity. T2DM accounts for approximately 90% of all cases of DM [5,6,7,8]. The pancreatic β cells are damaged, and the resulting massive inflammation, and glucose-derived reactive oxygen species eventually lead to insulin deficiency; these present substantial challenges in diabetes treatment and long-term prognosis [9,10,11]. To date, marketed anti-diabetic drugs are effective in regulating diabetes-associated high and fluctuating blood glucose levels, via improving the tissue sensitivity or increasing the available insulin, instead of protecting pancreatic β cells from free radicals, oxidants, and the subsequent inflammation and cell apoptosis [12,13]. Moreover, the risk of toxin accumulation at the gut level, and hypoglycaemia, are also major side effects common to many anti-diabetic drugs, which may compromise the clinical efficiency of the drug [11]. Long-term use of an anti-diabetic drug, such as sulfonylureas, causes β-cell apoptosis due to extreme cell stimulation [14]. Therefore, the use of anti-oxidants (as adjuncts) as potential therapies is based on their ability to protect pancreatic β cells from inflammation, free radicals and oxidation, is currently gaining significant interest in potential therapies for the treatment of T2DM [15,16,17].

Probucol (PB) is a highly lipophilic compound, classified as BCS class-II (Biopharmaceutics Classification System) drug, initially developed for the treatment of hyperlipidaemia [18]. PB is reported as a cardioprotective agent and it has been shown that PB-treated rats had markedly reduced myocardial ischemia lesions [19]. It was clarified that one of the causes of DM is due to oxidative stress and inflammation involved in the pathogenesis of islet lesions [20,21]. A study done by Gorogawa and his colleagues has shown that PB can exert anti-diabetic effects by protecting pancreatic β cell function in T2DM animal models [22]. Likewise, another similar study revealed that PB treatment in hamsters partially restored pancreatic β-cell function and decreased serum glucose levels [23]. PB restores insulin secretion by protecting the pancreatic β cells, which supports glucose haemostasis. [15,16,24]. Therefore, PB has great potential in treating diabetes because of its potent anti-inflammatory and antioxidant properties, and pancreatic β-cell shielding effectiveness. However, it failed to demonstrate consistent desirable effects due to its lipid solubility, three compartmental modelling, high volume of distribution and poor and variable bioavailability; hence, drastically robust orally targeted systems are necessary [19,24,25,26,27]. 

As formulation excipients, bile acids (BAs) can enhance absorption, act as a permeation-enhancing agent and facilitate the drugs’ uptake [28,29,30,31]. Our lab demonstrated through chambers diffusion studies that BAs, when incorporated via artificial cell microencapsulation technology (ACMT), exert beneficial anti-diabetic effects *in vivo* [32,33] and enhance drug uptake *ex vivo* [34,35,36]. BA derivatives have improved anti-diabetic drug absorption via ileum [35]. Findings showed that BA microcapsules prepared using ACMT improved PB oral absorption for T2DM animals [28,29,37]. However, PB’s absorption profile remained limited, and its release profile from the microcapsules remained variable. Therefore, we hypothesized that using a more structurally stable, rigid and less soluble BA may improve PB release patterns, and promote the targeted delivery by increasing water permeation in microcapsules. Unconjugated lithocholic acid (ULCA) is a promising compound, endogenously produced in humans, and recently it was shown to possess formulation-stabilizing and anti-inflammatory effects [38,39]. Moreover, ULCA has substantially different chemical structure compared with other Bile acids (BAs), different hydrophilicity, and contrasting effects *in vivo*. From other BAs, UDCA (ursodeoxycholic acid) is among the most studied ones. Compered to UDCA, ULCA has vastly deferent ligand potency, particularly *in vivo*, as ULCA is a much more potent ligand for several BA receptors (FXR-Farnesoid X Receptor and TGR5-Takeda G-Protein-Coupled Receptor-5) compared to UDCA, including VDR (vitamin-D receptor), which UDCA is not specific ligand for [40,41,42].

Recently, studies have come up with a new concept called “Design of Experiment” (DOF), to understand a drug’s biophysical properties and manufacturing processes, which is used in the formulation development of nanoparticles and microparticles. DOF is one important tool in employing quality by design in formulation development [43]. Latterly, the use of micro/nanoparticles has gained more attention in targeted drug delivery, showing high drug availability and treatment efficacy for many diseases such as cancer, skin disease, a neurodegenerative disorder, and inflammatory diseases [44,45,46,47]. Likewise, polymer-based micro/nanocapsules have a particular interest in controlled and targeted drug delivery [48]. The selection of the polymer is a vital step in drug delivery and discovery, as it affects drug release and absorption [49]. Sodium alginate (SA) has shown the most promising result in oral drug administration [31,50]. SA is a natural polysaccharide extracted from brown algae and has characteristic features of being biodegradable and biocompatible, and it has demonstrated excellent stability and compatibility, as well as pH-dependant degradation and release kinetics which are also influenced by its viscosity [51,52]. Specifically, low-viscosity SA (LVSA) showed controlled and targeted drug release in comparison with high-viscosity SA (HVSA), primarily when targeting the cecum [53]. In gastrointestinal (GI) fluid, the hydrated LVSA matrix changes into the porous, insoluble acid matrix and when it reaches higher pH values (the small intestine), the alginate acid matrix is transformed into a soluble viscous layer that breaks down the polymer integrity and releases drugs from the microcapsules [53,54]. Previously, our lab has demonstrated that sodium alginate-based formulation improved pharmacokinetics and pharmacodynamics responses with optimized structural, chemical, and physical compatibility and improved targeted delivery [31,55,56]. This study is a preliminary study to elucidate the application of our ionic gelation vibrational jet flow technology in nano/microcapsule production, before moving a step further toward an *in vivo* animal study of anti-oxidant for T2DM. Therefore, in this study, newly designed PB–LVSA and PB–ULCA–LVSA microcapsule formulation systems were evaluated as potential drug delivery systems in the management of T2DM, which can be extended to other lipophilic anti-diabetic drugs.

## 2. Materials and Method

### 2.1. Materials

PB (99.89%) was purchased from Medisca (Las Vegas, NV, USA), sodium alginate low viscosity (99%) and ULCA (≥95%) were purchased from Sigma-Aldrich CO., (St Louis, MO, USA). Calcium chloride dehydrates (CaCl_2_.2H_2_O, 98%) were purchased from Scharlab S.L (Sentmenat, Spain).

### 2.2. Drug Preparations

LVSA (1.2%), PB (4%) and ULCA (2%) stock suspension was prepared by the slow addition of powder in 8% water-soluble gel. CaCl_2_ (5% *w*/*v*) was made by adding CaCl_2_ powder in ultrapure milliQ water. Prepared formulations were thoroughly mixed for 6 h at room temperature, stored in the fridge, and used within 24 h of preparation.

### 2.3. Microcapsules Preparation

PB–LVSA (F1-without ULCA as control) and PB–ULCA–LVSA (F2-with ULCA as test) microcapsules were prepared using our established system (BÜCHI Labortechnik AG, Flawil, Switzerland) based on our developed technology: the ionic gelation vibrational jet flow technique [29,55]. Multiple parameters were used, including a frequency of 1800 Hz with a constant air pressure of 350 mbar, and a liquid flow rate of 5 mL/min; these parameters were constant for both microcapsules. The microcapsules were prepared with or without ULCA to the final concentration of PB:ULCA:LVSA in a ratio of 1 × 3 × 30, respectively [12]. This ratio was based on our previous studies [13,57]. Microcapsules were collected in and left for 5–10 min in the CaCl_2_ ionic gelation bath to preserve the spherical shape of the droplets. To dry microcapsules, the stability chamber (Angelantoni Environmental and Climatic Test Chamber, Massa Martana, Italy) was used and the weight of dried microcapsules was recorded.

Morphology, drug contents, microencapsulation efficiency, production yield, mean particle size, surface tension, conductivity, zeta potential, swelling, mechanical resistance, buoyancy, release patterns, stability, and the biological activity of each preparation were analysed in triplicates (*n* = 3) [58,59].

### 2.4. Characterization of Loaded Microcapsules 

#### 2.4.1. Morphological Analysis and Surface Characterization of Microcapsules 

Morphological characterization was accessed by using Nikon H550S optical microscopy (OM) and scanning electron microscope (SEM) (Neon 40EsB FIB-SEM; Zeiss, Oberkochen, Germany). For SEM, the microcapsules were freshly made, dried and mounted on a glass slide stub with double-sided adhesive tape and coated under vacuum in an argon atmosphere with 5 nm platinum before examination. Elemental distribution present on the microcapsules’ surface was analysed by energy dispersive X-ray spectrometry (EDXR) (INCA X-Act; Oxford Instruments, Abingdon, UK) [28].

#### 2.4.2. Drug Content, Production Yield, and Microencapsulation Efficiency

Dry microcapsules (2 g) were ground and dissolved in 200 mL phosphate buffer saline (PBS) pH 7.8. The suspension was stirred for 6 h, and one mL of the solution was transferred and diluted with phosphate buffer to 20 mL volume. Before analysis, the prepared solution was filtered through a 0.22 µm Millipore filter (Sigma-Aldrich CO., St Louis, MO, USA). The dissolved drug content concentration in the solution was measured with a UV spectrophotometer (Shimandzu UV–Vis spectrophotometer 1240, Kyoto, Japan) at 242 nm against the buffer blank [60]. To confirm the method’s accuracy and precision, a HPLC (high-pressure liquid chromatography) procedure was done as per our previous research [61]. The analysis was done in triplicate and calculated by our previously established formulae, as mentioned [62]:

(1) $$\mathrm{Drug}\;\mathrm{contents}\;\left(\%\right)=\frac{Calculated\;amount\;of\;PB\;in\;the\;microcapsules}{Total\;weight\;of\;microcapsules}\times 100$$

(2) $$\mathrm{Production}\;\mathrm{yield}\;\left(\%\right)=\frac{Total\;weight\;of\;the\;microencapules}{Total\;weight\;of\;polymer\;and\;drug\;solution}\times 100$$

(3) $$\mathrm{Microencapsulation}\;\mathrm{efficiency}\;\left(\%\right)=\frac{drug\;content\;}{Theoretical\;content}\times 100$$

#### 2.4.3. Electrokinetic Stability, Size Analysis, Surface Tension and Conductivity

Electrokinetic stability and particle size analysis were determined using the zeta sizer and mastersizer, respectively (Zetasizer 3000 HS and Mastersizer 2000, Malvern Instruments, Malvern, UK) [37,62]. Surface tension (ST) was measured using a tensiometer (Sigma 703). Conductivity was performed with the help of the conductivity meter (CDM230, Conductivity Meter, Radiometer Analytical SAS, Lyon, France) by calibrating with the potassium chloride (KCl) standard.

#### 2.4.4. Swelling and Mechanical Resistance Studies

The microcapsule swelling properties were calculated by placing 200 mg of microcapsules (F1 and F2) in 20 mL PBS. This study was conducted at four different pH values (1.5, 3, 6 and 7.8) and at two different temperatures (room temperature and 37 °C) over 6 h. The selection of pH values and temperatures was based upon our previous work [37,62]. Swollen microcapsules’ net wet weight was measured by weighing on a dynamic balance immediately after blotting them on the filter paper (Whatman #40). The microcapsule swelling index percentage was determined as previously described [63]. For mechanical resistance, briefly, 25 dry microcapsules from each formulation batch (F1 and F2) were placed in 20 mL PBS (pH 7.4) and oscillated at a frequency of 150 rpm for 24 h (Boeco Company, Hamburg, Germany). The mechanical resistance index was calculated as per our previously established protocols [62,64].

#### 2.4.5. Buoyancy Test

One hundred dried microcapsules were taken and placed in 200 mL of PBS (pH 7.8). The buffer was stirred for 6 h at a speed of 50 rpm at a temperature of 37 °C using USP dissolution apparatus 24, type II (Erweka, DT6, Langen Germany). The temperature was regulated through thermostats. Every hour, the number of floating microcapsules was counted and calculated as previously described [29].

#### 2.4.6. Drug Release Studies—*In Vitro* Dissolution Test

Two and a half grams of F1 and F2 microcapsules were weighed and suspended in 300 mL of simulated intestinal fluids (SIF), which contained the PBS of four different pH values of 1.5, 3, 6 and 7.8 at 37 °C. The sink condition was maintained throughout the assay time, and the dissolution medium was stirred at 200 rpm for 6 h [62]. The solution’s absorbance was measured every 30 min (2 mL of the solution was taken from the dissolution bath and replaced with the same amount of blank buffer to maintain the equilibrium condition throughout the assay). The 2 mL solution removed from the dissolution bath was placed into a 10 mL flask (sink conditions were maintained during this assay). Following this, the transferred solution was diluted with PBS to the volume and further centrifuged to clarify the solution. The amount of released drug in the solution was measured with a UV spectrophotometer at 242 nm against the PBS blank. Additionally, to exclude any interferences and to confirm that only the drug was being measured, microcapsules without PB (LVSA microcapsules) were also analysed at all four pH values.

#### 2.4.7. Physical Stability

The stability testing of F1 and F2 was conducted by putting 25 freshly prepared microcapsules on microscope slides and storing them for four days under thermostatically controlled ovens at four different temperatures (−20 °C, 5 °C, 25 °C, and 40 °C) with relative humidity set at 35% in the stability chamber [62,64]. After four days, the microcapsules were observed under the OM for morphology and appearance change, then the drug content and release profile were calculated as mentioned above [62].

#### 2.4.8. NIT-1 Pancreatic β Cells and Biological Analysis

The pancreatic β-cell line (NIT-1) were kindly provided by Professor Morahan (The University of Western Australia) and cultured in Dulbecco’s Modified Eagle medium (DMEM) (Sigma-Aldrich, St Louis, MO, USA) with supplemented 10% bovine serum (Thermo Fisher Scientific, Melbourne, Victoria, Australia), and 5.5 mmol glucose (Sigma-Aldrich, St Louis, MO, USA). The cells were incubated in a humidified atmosphere of 5% CO_2_ at 37 °C. The media was changed at 48 h intervals, and the cells were sub-cultured when confluence reached 80% as per the standard method [65].

Cytokine release from NIT-1 cells was performed to examine the biological efficiency of the prepared microcapsules. For this, NIT-1 cells were cultured in the prepared media, DMEM (pH 7.4) at two different glucose concentrations (5.5 mmol and 25 mmol) and treated with F1 and F2 microcapsules. The microcapsules were put directly in the cells containing wells, and incubated together with the cells for 48 h. After 48 h, IFN-γ (interferon gamma) and IL-10 (interleukin 10) were examined by removing the treated microcapsules and analysing the cell media via the cytokine bead array flow cytometry analysis (BD Bioscience cytometry Bead Array Mouse, USA) with the cell analyzer BD FACSCanto II (BD Bioscience, USA). FlowJO software (FlowJo, Ashland, Oregon) was used to interpret data.

For the seahorse analysis, the cells were treated with microcapsules (F1 and F2) at 5.5 and 25 mmol glucose concentrations for 48 h. The assay was determined via a Seahorse Flux Analyser XF 96 (Seahorse Bioscience Billerica, MA, USA) standard [37].

#### 2.4.9. Statistical Analysis

Student’s *t*-test was performed to analyse the drug content, production yield and microencapsulation efficiency and the values are presented as mean ± SD where *n* = 3. Correlation, regression and one-way ANOVA/ two-way ANOVA (analysis of variance) were done to analyse the data and Tukey HSD post-hoc comparison of means was conducted when the data were statistically significant. GraphPad Prism version X8.2 (Graphpad, Inc., USA) was used for all analyses, and the data were considered of statistical significance at *p* < 0.01 or *p* < 0.05.

## 3. Results and Discussion

### 3.1. Microscopic Examination and Surface Analysis

**Figure 1:**
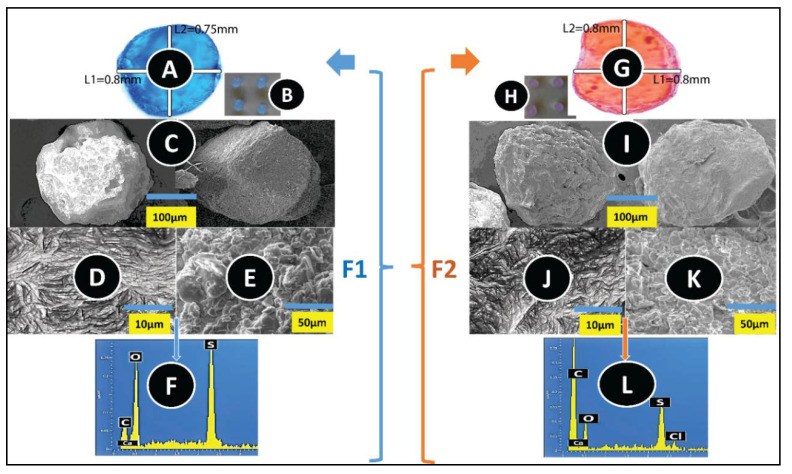
Morphological examination of microcapsules. (**A**,**B**,**G**,**H**) Optical microscopy. (**C**,**I**) Scanning electron micrographs. (**D**,**E**,**J**,**K**) Energy-dispersive X-ray spectra and figures (**F**–**L**) with the corresponding spectra. (F1): PB–LVSA microcapsules and (F2): PB–ULCA–LVSA microcapsules. PB—probucol; LVSA—low-viscosity sodium alginate; ULCA—unconjugated lithocholic acid.

The OM images showed (Figure 1A,G) that both the F1 and F2 microcapsules preserved their spherical shape, uniformity, and that the incorporation of ULCA did not alter the shape and size of microcapsules, which was further ascertained by the SEM images (Figure 1C,I). The average horizontal (L1) and vertical (L2) mean diameter of both microcapsules was 0.7 ± 0.1 mm. Our previous finding showed that microcapsules, with sizes ranging from 0.7 to 0.9 mm, supported the targeted gut release, improved cell viability, and had an hyperglycaemic effect on diabetic animals [31]. The surface topography of F1 (Figure 1D,E) and F2 (Figure 1J,K) presented rough and solid granules on the surface. Furthermore, corresponding spectra analysis on the microcapsules’ surface showed the abundance amount of C, Ca, O, and S, which suggest that ULCA did not adversely affect the microcapsule morphology and surface topography. The presence of S in the analysed spectra indicates that the granules on the surface are due to PB deposition, since none of the excipients contain S except PB; as the PB chemical formulation contains two S atoms (Figure 1F,L) [18]. The presence of Ca^2+^, C, and O was expected because they are part of the polymer used, and the ionic gelation bath, which was one of the prerequisites of the process during the manufacture of microcapsules [66]. The results support our previous studies that the incorporation of BAs did not change the shape, size, and surface drug content of PB microcapsules, which proved that the microencapsulation method is robust and uniform, regardless of any formulation [28,31,67].

### 3.2. Drug Content, Microencapsulation Efficiency, Production Yield, Zeta Potential, Size Analysis, Surface Tension, and Conductivity

**Figure 2:**
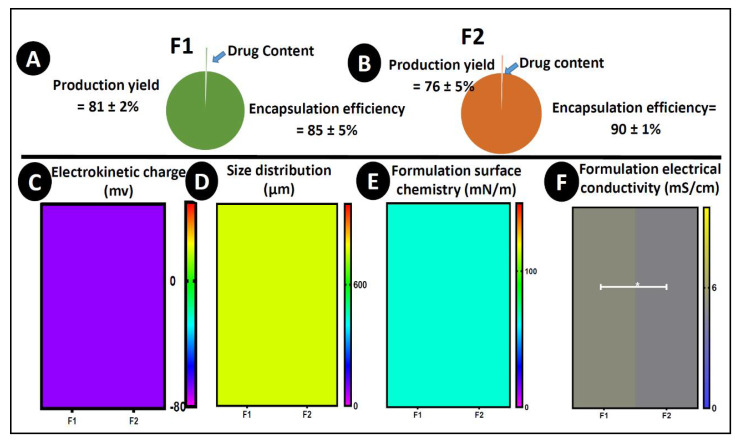
Drug content, production yield, and encapsulation efficiency (**A**,**B**), (**C**) electrokinetic charge, (**D**) size analysis, (**E**) surface tension and (**F**) conductivity. *N* = 3, mean ± SEM. F1: PB–LVSA microcapsules and F2: PB–ULCA–LVSA microcapsules. PB—probucol; LVSA—low-viscosity sodium alginate; ULCA—unconjugated lithocholic acid. * *p* < 0.01.

As shown in Figure 2A,B, the amount of PB content in both formulations (F1 and F2) remained constant with little variation but not statistically significant (F1 = 2.3 ± 0.2% and F2 = 2.27 ± 0.32%) (*p* > 0.05), which proved that the integration of ULCA in F1 did not alter the drug content of the microcapsules. The total manufacture yield and microencapsulation efficiency of F1 and F2 ranged from 70 to 92% and was not significantly different between the F1 and F2 microcapsules. A good level of PB loading around 85–90% was recorded for both kinds of microcapsules. This proves that the addition of ULCA has the least effect on the drug content, ability, and yield in both F1 and F2 microcapsules, which lines up with our previous lab studies (*p* > 0.05) [28,31].

The measurement of surface charge provides particle colloidal suspension properties, showing which kind of interaction the particles may have, whether they aggregate or disperse in solution. Usually, the higher the negative charge, the better the electrokinetic stability of the particles [68]. Likewise, the surface chemistry provides information about the formulation’s nature. With higher surface chemistry, the formulation is homogenous and smooth, with impacts on drug release kinetics [69]. Figure 2C–F shows that the surface charge (−60–70 mV) (*p* = 0.1167), size distribution (750–770 μm) (*p* = 0.8411), and surface chemistry (64–67 m/Nm) (*p* = 0.2931) remained constant after the addition of ULCA, suggesting that the dispersion of microcapsules was stable, and capable of withstanding particle agglomeration and flocculation [70]. However, the conductivity was diminished after mixing the ULCA in the PB–LVSA formulation (*p* < 0.01). The conductivity of the formulation provides information on the nature of the main vehicle in terms of the current conductivity being predominantly water-based; hence, the potential micelle formation and clear hydrophilic–lipophilic balance are significantly influenced by water. The addition of BAs increases the negative charge due to the removal of a proton from the carboxylic acid group, resulting in a net decrease in positive surface charge in the mixture, which decreases the conductivity of the formulations that support the kinetics of encapsulated drug release [71].

### 3.3. Swelling Index

**Figure 3:**
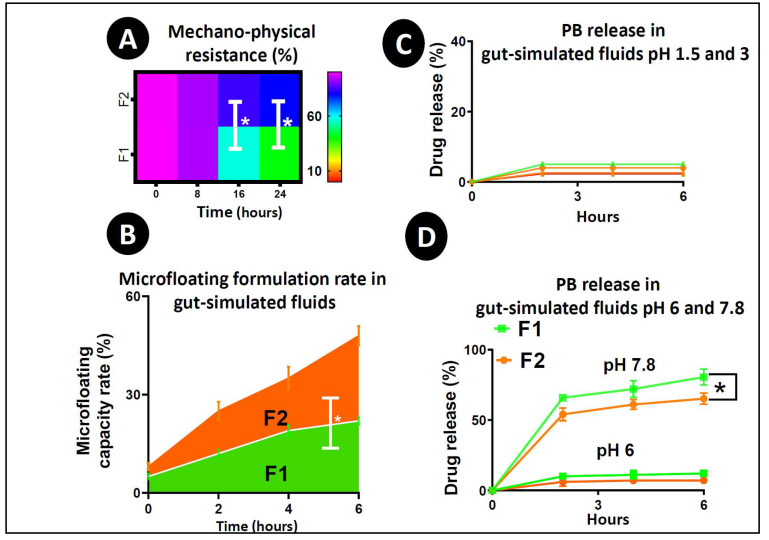
(**A**) Microcapsules mechanical strength testing; control (F1) and test (F2); (**B**) microcapsules buoyancy index. (**C**,**D**) Microcapsules dissolution profiles in simulated gastric media at pH 1.5, pH (**C**); pH 6, pH 7.8; (**D**). *N* = 3, mean ± SEM. F1: PB–LVSA microcapsules and F2: PB–ULCA–LVSA microcapsules. PB—probucol; LVSA—low-viscosity sodium alginate; ULCA—unconjugated lithocholic acid. * *p* < 0.05.

Table 1 incorporates the swelling index of F1 and F2 microcapsules. The swelling test was performed at four different pH values (1.5, 3, 6, and 7.8) and two different temperatures (25 °C and 37 °C). Table 1A,B show that the temperature and pH of the medium affect the swelling properties of the microcapsules. The addition of ULCA in F1 significantly decreased the swelling behaviour of the microcapsules (*p* < 0.01) at high pH and temperature compared to low pH and temperature, suggesting a better control of PB release in the target site of the intestine at pH 7.8; which is further complemented by a mechanical resistance and buoyancy test (Figure 3A,B). Usually, alginate undergoes extensive swelling at higher temperatures and pH because of the higher water uptake, an increase in porosity and the solubilisation of the polymer [52]. In addition, heat often causes the erosion and breakdown of the microcapsule wall (matrix wall), which allows for more significant water infiltration [72]. This result undoubtedly backed our hypothesis that BAs enhance membrane stabilization by cross-linking properties or ionic interaction with the alginate matrix [33,34,35].

### 3.4. Mechanical Strength, Buoyancy Test and Drug Release Studies

Mechanical resistance provides ideas about the microcapsules’ ability to resist mechanical stress and pressure. Figure 3A shows the mechanical index of the microcapsules in a percentage over a 24 h time frame. Until 12 h, there was no statistical alteration in the number of intact microcapsules between the control and test. However, after 12 h, the control microcapsules started losing their shape and dry contents, as well as physical adherence. After 16 h, almost 50% of the F1 microcapsules became deformed, while 80% of the F2 microcapsules remained intact (*p* < 0.05); which proved that the addition of ULCA significantly enhanced the microcapsule’s strength and can prevent premature drug loss due to a rapid change in GI pH values (1.5–7.8) when administrated orally [29,31,64].

Figure 3B shows the *in vitro* buoyancy test of the control (F1) and test microcapsules (F2) over 6 h. In the buoyancy test, the percentage of floating microcapsules is calculated over a time period. At the end of 6 h, the portion of floating microcapsules for F1 was below 30%, while part of the floating microcapsules was almost 50% for F2 microcapsules (*p* < 0.05). Improved F2 buoyancy proves that microcapsules could maintain this property in the stomach and support and optimize the controlled and targeted release of the carrier drug. Overall, our swelling study findings from Table 1, including mechanical resistance (Figure 3A) and *in vitro* buoyancy test (Figure 3B) prove that the incorporation of ULCA into F1 microcapsules leads to more stable microcapsules by supporting membrane integrity and physical coherence. The release study (Figure 3C,D) complements the above results and endorses that the addition of ULCA results in more coherent and more potent microcapsules at a high pH value of 7.8, which emphasises its ability to withstand degradation in the lower GI tract, resulting in higher controlled release and promote targeted drug delivery. These results were consistent with our previous studies [29,73].

Figure 3C,D show the PB release at 1.5, 3, 6, and 7.8 pH over 6 h at a temperature of 37 °C for two different formulations (F1 and F2). These pH values were taken based on the sites of anti-diabetic drug absorption in the GI and pH gradient system [28,62,73]. The results show that the release of PB is mainly dependent upon the pH and formulation type. At lower pH values (1.5, 3 and 6), the alginic acid present in the LVSA matrix usually leads to shrinkage, which helps the encapsulated drug to remain within the core of the microcapsules. Still, at a higher pH (>6) values, due to the quick dissolution and solubilisation, alginic acid forms a soluble viscous layer [74], resulting in the microcapsule bursting and the subsequent release of the encapsulated drug.

As seen in Figure 3C, at pH 1.5, there was a low drug release of only 2–2.5%, and 3–5% at pH 3 from both F1 and F2 microcapsules. As predicted, the release of PB was higher at pH values of 6 (5–10%) and 7 (60–80%) (Figure 3D). Notably, the release pattern was significantly higher at pH 7.8 in comparison with pH 6 for both formulations (*p* < 0.05), which coincides with the targeted delivery in the distal site of the intestine. After 6 h, the drug release from F1 microcapsules at pH 7.8 reaches up to 80%; whereas the drug release from F2 microcapsules peaks at 54–65% (*p* < 0.05). This feature is important in the development of diabetes therapy, as most of the anti-diabetic drugs are absorbed from the distal site of the intestine (pH 7–7.8) [74]. Other studies presented different and inconsistent effects of drug release such as the biphasic, multiphasic and rapid burst of drug when other BAs were used to formulate microcapsules [28,62]. In contrast, this study shows the continuous and targeted release of the PB at pH 7.8, probably because of the structural, physical and chemical nature of ULCA. This study further supports that targeted drug release from BA microcapsules depends upon the formulation. Compared to other BAs, ULCA is less soluble but has excellent excipient and stability properties, which assist in resisting dissolution even if the pH values are higher. This is achieved by retaining cross-links with LVSA, which results in increased membrane stability, and in turn, protects the microcapsule from rapid degradation and allows for a more controlled release of encapsulated PB [75,76] as seen in Figure 3A,B and Table 1. Moreover, the EDXR results (Figure 1) showed the potential presence of PB on the surface, as well as the inside of the capsules, which is expected and similar to previously published data on drug encapsulation using BA-based capsules [29]. This can allow for a more controlled and prolonged drug release profile, since initial release can take effect immediately in the intestine, followed by a more slowed release. The findings are consistent with previously published data, this release profile suggests the successful incorporation of ULCA within the designed capsules, as well as the even distribution throughout the layers, including on the surface. Therefore, the PB release from the ULCA-based microcapsules showed a targeted release and revealed the most favourable characteristics for anti-diabetic drug delivery. The PB release was conducted in SIF in different pH values. In the future, the release patterns will teste in an *in vivo* murine model to reflect and compare different fluid dissolution behaviour; fasted state simulated intestinal fluid (FaSSIF), fasted state simulated gastric fluid (FaSSGF), fed state simulated intestinal fluid (FeSSIF) and fed state simulated gastric fluid (FeSSGF).

### 3.5. Stability Studies

**Figure 4:**
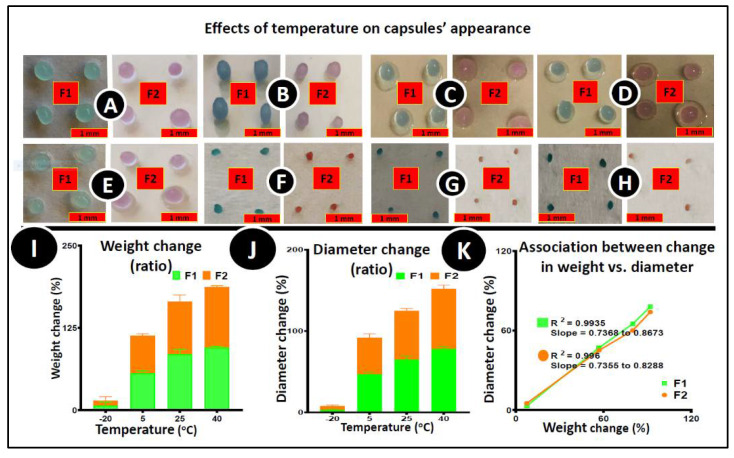
Effect of temperature on the microcapsules’ appearance. Pictures were taken before (**A**–**D**) and after the accelerated stability testing (**E**–**H**); (**I**) % change in weight, (**J**) % change in diameter and (**K**) correlation between % changes in diameter and % change in weight. *N* = 3, mean ± SEM. F1 (control): PB–LVSA microcapsules and F2 (test): PB–ULCA–LVSA microcapsules. PB—probucol; LVSA—low-viscosity sodium alginate; ULCA—unconjugated lithocholic acid.

Figure 4 shows the microcapsules’ (control and test) morphological characteristics before (Figure 4A–D) and after (Figure 4E–H) the accelerated stability testing at various temperatures; −20°C (Figure 4A,E), 5 °C (Figure 4B,F), 25 °C (Figure 4C,G) and 40 °C (Figure 4D,H) over 96 h at 35% humidity. Both F1 and F2 microcapsules successfully preserved their original morphological characteristics (shape) during the study period at a lower temperature (−20 °C and 5 °C). Both microcapsules’ size and weight decreased drastically with increasing temperatures (25 °C and 40 °C) (Figure 4I,J) due to the water content evaporation from the microcapsules [62]. Figure 4K showed a positive correlation between the size of the microcapsules and the weight changes. More than a 50% reduction in size, and a 70% reduction in weight of the microcapsules was found, and the most significant effect was seen at a temperature of 40 °C. The microcapsules became brittle and hard, and lost elasticity on all temperatures except −20 °C. Due to the microcapsules’ moisture content evaporation, many more changes were noticed at 40 °C, possibly because of the potential oxidation and dehydration effects on the microcapsules. After four days, the UV–Vis spectrophotometric analysis was conducted to establish the drug content (post-stability study), concluding that there were no significant changes in drug content. The average percentage of drug content for PB–LVSA was 2.21 ± 0.1, and 2.32 ± 0.2 for PB–ULCA–LVSA, respectively. Similarly, the drug release pattern after the post stability study was conducted in two different pH values (6 and 7.8) and the result showed that the drug release was higher in pH 7.8, and the release pattern was continuous and controlled in the presence of ULCA, similar to the freshly prepared microcapsules as presented in Figure 3C,D (data are not shown). The post-stability drug content was similar to fresh microcapsules, which proved that there was no significant loss of PB under these testing conditions from both microcapsules. This study is consistent with a previous study where the F1 and F2 microcapsules’ physicochemical compatibility was analysed [39]. Differential scanning calorimetry (DSC) results showed that PB did not participate in a cross-linking reaction with LVSA and BA, and did not compromise thermal and chemical integrity during the microencapsulation process, which is further supported by the Fourier-transform infrared spectroscopy (FTIR) analysis [39]. The addition of ULCA has no impact on the stability of PB microcapsules. Thus, the stability study confirmed the uniformity of the PB content with no noticeable differences from changing temperatures and conditions.

### 3.6. Biological Activity of PB-Loaded Microcapsules

#### 3.6.1. Pancreatic β-Cell Cytokine Measurement

**Figure 5:**
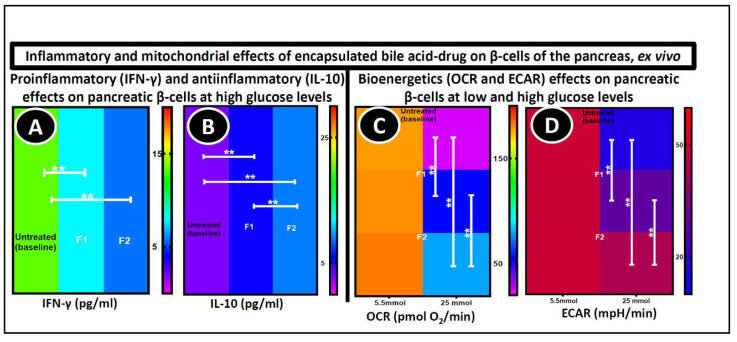
Cytokine production test (**A**,**B**) 48 h post-treatments and bioenergetics parameters (**C**,**D**) using the NIT-1 pancreatic β cell lines at two glucose concentrations: 5.5 mmol and 25 mmol. *N* = 3, mean ± SEM. F1: PB–LVSA microcapsules and F2: PB–ULCA–LVSA microcapsules. PB—probucol; LVSA—low-viscosity sodium alginate; ULCA—unconjugated lithocholic acid; IFN-γ—interferon gamma; IL-10—interleukin 10; OCR—oxygen consumption rate; ECAR—extracellular acidification rate ** *p* < 0.01.

Plasma biomarkers like IFN-γ have been linked with inflammation and expressed widely in patients with diabetes [77]. Their expression level is interconnected with the development and progression of diabetes. In Figure 5A,B, the level of expression of IFN-γ and IL-10 were measured in NIT-1 pancreatic β-cells exposed to hyperglycaemia (25 mmol) and treated with F1 and F2 microcapsules for 48 h. The cytokines expression in the NIT-1 cells after microcapsule treatment was compared to the cells treated with empty microcapsules. Figure 5A shows that the level of IFN-γ production was significantly higher in untreated cells (13.50 ± 0.90 pg/mL) (*p* < 0.01), whereas the levels decreased in cells treated with microcapsules (F1 = 7.90 ± 1.4 pg/mL and F2 = 5.80 ± 1.30 pg/mL). The basic MTT assay was initially carried out to examine the cell biological activity and the results were consistent with IFN-γ levels [39]. Likewise, the expression of anti-inflammatory cytokine IL-10 was 3 ± 0.80 pg/mL in the control and increased considerably in the treated cells (F1 = 5.70 ± 0.85 pg/mL and F2 = 8.90 ± 0.72 pg/mL) (*p* < 0.01) (Figure 5B). The potent anti-inflammatory effect of PB that supports cell proliferation, reduces apoptosis signalling, and increases the expression of anti-inflammatory and decreases the level of pro-inflammatory cytokines has been previously described [9,78]. Interestingly, the addition of ULCA in PB microcapsules significantly increased the expression of the IL-10 cytokine (*p* < 0.01). This suggests that ULCA also may have a decisive role in cell proliferation and support the expression of anti-inflammatory cytokine production. This is due to the stabilizing properties of ULCA, as presented in Table 1 and Figure 3A, which showed that ULCA act as a stabilizing effect on the microcapsules, thereby reducing the swelling property and improving the microcapsules mechanical resistance [22,79]. However, no significant difference was noted between the F1 and F2-treated cells in IFN-γ expression, possibly because of the different metabolic and secretory pathway compared to other cytokines [80]. The reduction in IFN-γ and enhancement in IL-10 that the PB–ULCA microcapsules showed, are expected to decrease cell apoptosis and support cell function, which is supported by bioenergetics analysis [81]. Previously published data have displayed that the anti-oxidant activity of BAs enhanced β-cell viability [39,81,82,83].

#### 3.6.2. Seahorse Analyses

Different parameters, such as the oxygen consumption rate (OCR) and extracellular acidification rate (ECAR), which assess mitochondrial function, were measured and presented in Figure 5C,D. NIT-1 cells were treated with empty microcapsules (control), F1, and F2 microcapsules for 48 h at two glucose concentrations (5.5 mmol and 25 mmol glucose). This shows that there is no cell stress at 5.5 mmol, which causes no significant changes in the cellular metabolism biomarkers and bioenergetics parameters between the control and test (F1 and F2) cells. At 25 mmol, due to the cell stress, significant changes in the OCR and ECAR were found between the untreated and treated cells (*p* < 0.01). The ULCA incorporation in the F1 microcapsules significantly improved the β cells OCR (from 57 ± 12 to 79 ± 19 pmol O_2_/min) (*p* < 0.01) (Figure 5C) and ECAR (29 ± 3.9 to 44 ± 5.2 mpH/min) level (*p* < 0.01) (Figure 5D). This significant improvement suggests a strong positive influence of the PB-loaded microcapsules on the β-cells’ biological activity, such as glycolysis and mitochondrial respiration [84,85]. New oxygen molecules are generated by electron acceptors, stimulating an oxidative phosphorylation chain to synthesize ATP (adenosine triphosphate), and ultimately increase insulin secretion from β-cells. However, one major limitation of this study is the lack of insulin secretion data. Different studies also showed that PB-loaded microcapsules had anti-oxidant and anti-inflammatory properties that improve mitochondrial respiration and metabolic activity [37,67,86]. In recent literature, it was shown that PB-ursodeoxycholic acid microcapsules have similar effects on cellular parameters, viability and drug release, proving that these effects were formulation dependent [81]. From these biological results, the F2 microcapsules undoubtedly showed a positive and significant protective effect on pancreatic β-cells, and improvement in bio-energetic parameters.

Due to the focus of the paper on the effect of ULCA on PB release, biological changes were examined using two different microcapsules, one with ULCA and one without ULCA. Untreated cells were considered as control. Future studies will endeavour to examine the biological impact of ULCA alone, using ULCA novel delivery matrices. Overall, to our knowledge, this is the first preliminary study of the application of ionic gelation vibrational jet flow technology in producing microcapsules with an encapsulation efficiency ≥90% as the result of ULCA incorporation, with empowered surface chemistry and electrical conductivity. Furthermore, the findings illustrate a clear association between the change in weight vs. diameter and the clear impact of ULCA integration on the most powerful anti-inflammatory cytokine.

## 4. Conclusions

Our microencapsulation method with set parameters produced excellent and uniform microcapsules. The integration of ULCA on F1 microcapsules did not affect the size, shape, uniformity, stability and microcapsule drug content, but enhanced the microcapsules strength. Furthermore, the ULCA addition leads to more stable microcapsules by reduces swelling, and allowing for controlled and pH-targeted drug release. The ULCA incorporation enhanced bioenergetics parameters, decreased the inflammatory cytokines and increased the anti-inflammatory cytokines. Overall, this suggests the potential applications of PB in the oral administration in T2DM. Future studies will endeavour to evaluate the pharmacology of the microcapsules *in vivo* using diabetic rodent models.

## Figures and Tables

**Table 1:** Swelling index at pH 1.5, 3, 6 and 7.8 for 25 °C (A) and 37 °C (B). *N* = 3, mean ± SEM. F1: PB–LVSA microcapsules and F2: PB–ULCA–LVSA microcapsules. PB—probucol; LVSA—low viscosity sodium alginate; ULCA—unconjugated lithocholic acid. * *p* < 0.01.

**Temperature = 25 °C (A)**				
**Formula Code**	**pH 1.5**	**pH 3**	**pH 6**	**pH 7.8**
**F1**	0.92 ± 0.005	1.873 ± 0.0625 *	3.286 ± 0.148 *	3.90 ± 0.11 *
**F2**	0.89 ± 0.005	1.383 ± 0.343 *	2.633 ± 0.104 *	3.08 ± 0.05 *
**Temperature = 37°C (B)**				
**F1**	0.99 ± 0.005	2.345 ± 0.005 *	3.83 ± 0.056 *	4.89 ± 0.095 *
**F2**	0.933 ± 0.057	2.12 ± 0.081 *	2.986 ± 0.349 *	3.87 ± 0.161 *
